# Human bornavirus research: Back on track!

**DOI:** 10.1371/journal.ppat.1007873

**Published:** 2019-08-01

**Authors:** Dennis Rubbenstroth, Kore Schlottau, Martin Schwemmle, Jürgen Rissland, Martin Beer

**Affiliations:** 1 Institute of Diagnostic Virology, Friedrich-Loeffler-Institut, Greifswald - Insel Riems, Germany; 2 Institute of Virology, Medical Center - University of Freiburg, Freiburg, Germany; 3 Faculty of Medicine, University of Freiburg, Freiburg, Germany; 4 Institute of Virology/Public Health Laboratory Saarland - University Medical Center, Homburg, Germany; University of Wisconsin Madison, UNITED STATES

Bornavirus infections in man have been a matter of controversial scientific debate for most of the last three decades. Because Borna disease virus 1 (BoDV-1) was known to cause fatal encephalitis and behavioral changes in a broad range of infected animals, it is perhaps not surprising that some virologists and clinicians suspected bornaviruses to also induce behavioral and psychiatric disorders in people. In contrast, the possibility of lethal bornavirus-induced disease of the human central nervous system was not appropriately taken into consideration. As recent research shows, however, for years they have been barking up the wrong tree.

## Global interest in an unusual animal pathogen

BoDV-1 (originally abbreviated BDV) was initially identified in a rather small area of Western Europe where domestic mammals such as horses or sheep were found to die of an encephalitis called “Borna disease.” BoDV-1 exhibits a remarkable neurotropism in most permissive species. Experimentally, it can infect a broad range of mammalian and even some avian species, in which it usually causes severe neurologic disorders resembling those observed in naturally infected horses and sheep. However, in certain models, rather subtle behavioral changes occurred in the absence of encephalitis [1–3]. Based on these intriguing observations and the broad experimental host range, it was soon proposed that the virus may also cause behavioral abnormalities in humans, and this not only in the endemic areas in Europe but perhaps globally [1, 4].

## Misleading presumptions: BoDV-1 as cause of psychiatric disorders

And so the race was on to demonstrate the presence of the virus in people with depression and other psychiatric or behavioral disorders. Rott and colleagues were the first to report bornavirus-reactive antibodies in sera from patients in Germany and the United States [4], and similar findings were soon published by other groups [1–3]. Given its potential clinical significance, bornavirus research flourished, rapidly leading to the molecular characterization of the virus and the analysis of its T lymphocyte–mediated immunopathogenesis in experimental rodent models [1–3]. There were some flies in the ointment, though: BoDV-1–reactive antibodies were also found in the blood of healthy donors worldwide who had no history of psychiatric disorders, and so were viral antigens, antigen–antibody complexes, and BoDV-1 RNA [1–3, 5]. Other studies, however, were unable to detect any signs of infection, regardless of whether they dealt with apparently healthy or sick people [1–3, 6]. Nevertheless, the overall conclusion was that BoDV-1 represented a true human pathogen that circulates in healthy humans worldwide and that occasionally causes psychiatric and behavioral disorders [1–3, 5].

This view, however, was soon disputed. First, it was argued that, in contrast to the findings in humans, the telltale sign of a BoDV-1 infection in animal hosts was a severe and often fatal encephalitis, and that clinically confirmed animal infections occurred only in a small endemic area in Europe [1–3]. Second, there were suspicions about the credibility of the diagnostic tests employed, as they were not standardized and often not reproducible in other laboratories. BoDV-1 is known to be easily detectable in the brains of diseased horses and sheep but not in their blood or peripheral organs [1–3]. Yet, most human studies focused on blood, as brain tissue was rarely available [1–3, 5]. Because viral loads in blood were expected to be extremely low compared with those in brain, detection methods were understandably tailored towards maximum sensitivity and not necessarily specificity. These tests included a unique enzyme-linked immunosorbent assay (ELISA) to detect BoDV-1 antigen and so-called “circulating immune complexes” [5], as well as nested reverse transcription polymerase chain reactions (RT-PCR) that were highly sensitive but also prone to laboratory contamination and false positive results [1, 7]. The techniques’ drawbacks came to light when the first phylogenetic analyses of BoDV-1 sequences showed that, while BoDV-1 sequences from the brains of diseased animals in the endemic area clustered according to their region of origin, the supposedly human sequences did not [7, 8]. Instead, virtually all of these human sequences were largely identical with the sequences of the laboratory strains used as positive controls in the respective institutions, thereby strongly suggesting contamination [7, 8]. In effect, these findings led to a shift away from the concept of widespread human BoDV-1 infections, and by 2010, human bornavirus research had essentially been halted in many of the groups that had previously been active in this field.

## Unraveling the mystery of bornavirus biology and epidemiology

**Fig 1:**
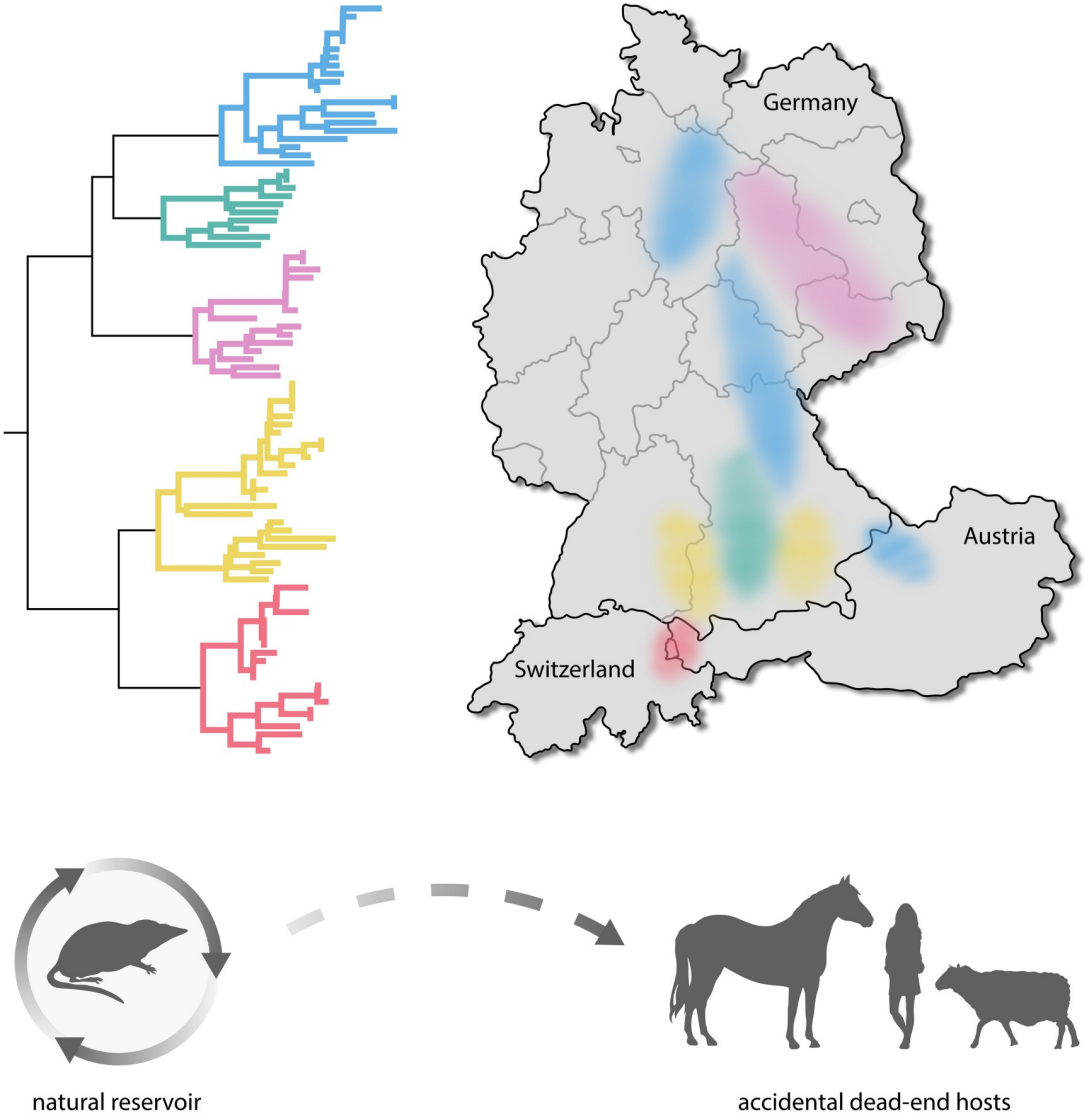
Occurrence and epidemiology of animal and human BoDV-1 infections. Genetically, BoDV-1 variants segregate into five distinct clusters (represented by different colors in the phylogenetic tree). Each cluster appears to be bound to confined regions in Germany, Switzerland, and Austria, where the viruses are maintained in infected bicolored white-toothed shrews (*C*. *leucodon*) serving as their natural reservoir. Persistently infected reservoir hosts stay apparently healthy and maintain the infection chain by shedding infectious virus. Accidental BoDV-1 transmissions to horses, sheep, humans, and other mammals result in often fatal immune-mediated encephalitis. In striking contrast to the reservoir host, the virus is restricted to the central nervous system in these accidental dead-end hosts that, thus, do not contribute to the spread of the virus. BoDV-1, Borna disease virus 1.

The above phylogenetic analyses not only identified the suspected human BoDV-1 sequences and the few supposedly human isolates as laboratory contaminants, but also helped to better understand the epidemiology of Borna disease in animals and its remarkable restriction to a small endemic area. Clearly, there were distinct and nonoverlapping regional sequence clusters within the endemic area (Fig 1). This striking pattern suggested that BoDV-1 was not transmitted between horses and/or sheep but instead originated from an at that time unknown, less mobile and strongly territorial reservoir, such as a rodent or another small mammal [9]. Screening of small mammals in endemic regions finally led to the detection of BoDV-1 in bicolored white-toothed shrews (*Crocidura leucodon*) [10]. Infected bicolored white-toothed shrews apparently stay healthy and show no signs of neural inflammation, despite a broad range of infected tissues, viral shedding in urine, feces, and saliva, and probably lifelong virus persistence [11]. These characteristics make them an ideal natural viral reservoir (Fig 1). In contrast, as mentioned above, in domestic mammals such as horses and sheep, BoDV-1 infection is strictly neurotropic and induces immune-mediated encephalitis [1–3]. It was conceivable, therefore, that domestic animals represent accidental dead-end hosts that do not contribute to the spread of the virus (Fig 1). By the same token, humans may likewise be spillover hosts. If so, infected humans should be found predominantly in the endemic area and show signs of encephalitis.

## True human bornavirus infections resemble Borna disease in animals

In 2015, variegated squirrel bornavirus 1 (VSBV-1), a close relative of BoDV-1, was discovered in healthy exotic squirrels kept as pets in European holdings [12]. Surprisingly and sadly, at least four breeders and caretakers came down with lethal encephalitis apparently associated with infection by this novel virus [12, 13]. Thus, similar to BoVD-1, VSBV-1 does not seem to harm its reservoir host but might cause fatal Borna disease-like encephalitis upon transmission to other species. These alarming findings once again changed the view on the zoonotic potential of bornaviruses and prompted some clinicians to consider them in patients with severe encephalitis of unclear origin.

This search was successful. Only three years after the discovery of VSBV-1, two independent groups in Germany simultaneously discovered the first human BoDV-1 infections in a total of five patients with encephalitis [14–16]. Three of the patients had received an organ transplant from the same donor [14], while in two independent cases, previously healthy young persons were affected [15, 16]. All five cases were characterized by nonsuppurative encephalopathies that resembled Borna disease in horses and were fatal in three of the patients. In three patients, the disease included polyradiculoneuritis and started with the clinical presentation of Guillain-Barré syndrome [14, 16]. In contrast to the previously suspected “human bornavirus infections,” viral antigen and high levels of viral RNA were detected in the brains of all three deceased patients, and high titers of bornavirus-reactive antibodies were present in sera and cerebrospinal fluids. The organ donor and both independent patients originated from different endemic regions in Bavaria, Southern Germany. Whole-genome sequencing revealed that their viruses were most closely related to isolates obtained from horses, sheep, and shrews of the same regions [14–16]. This is in good agreement with the view that humans, like horses and sheep, represent accidental dead-end hosts that can develop Borna disease-like encephalitis following BoDV-1 transmission from the local reservoir.

## Bornavirus-induced human encephalitis: Clinical alertness is required

The recent cases demonstrate that not only VSBV-1 but also BoDV-1 is a zoonotic human pathogen associated with fatal encephalitis. Thus, bornavirus infections in humans cause a clinical picture that is strikingly different from that suggested three decades ago. Remarkably, all diagnostic tools for detecting BoDV-1 had been available already in the 1990s, with the sole exception of next-generation sequencing. True human BoDV-1 infections could have been discovered 20 years earlier, if scientists had studied brain samples from severe encephalitis cases occurring in the known endemic regions instead of focusing globally on psychiatric patient cohorts. The developments in this field thus teach us the important lesson that, to guard against blind alleys, it helps to have a good theoretical framework that fully integrates the available evidence, in this case including epidemiological and clinical data.

So what is in store for the future? Research evidently needs to expand into retrospective and prospective studies within the BoDV-1 endemic area to shed light on the incidence of human BoDV-1 infections, their pathogenicity, and the direct or indirect transmission routes from the wild reservoirs. A particularly challenging task is the improvement of tools for reliable intravitam diagnostic strategies without repeating the shortcomings of the past. In addition to BoDV-1 and VSBV-1, the diagnostic screening may also include their closest relative Borna disease virus 2 (BoDV-2), as it was detected in an encephalitic horse in Southern Austria [17]. The examples of BoDV-2 and VSBV-1, as well as the growing number of known avian and reptilian bornaviruses, indicate that further mammalian bornaviruses may remain to be discovered in the future. Worldwide research should therefore aim at identifying their natural reservoirs and dispersal areas. The careful use of national and international notification regulations is mandatory in assessing their zoonotic potential and the risk of human exposure and disease.
